# 

**Published:** 1909-10

**Authors:** 


### BOOKS RECEIVED.

A Textbook of Protozoölogy. By Gary N. Calkins, Ph.D., Professor of Protozoölogy in Columbia University, New York. Octavo, 349 pages, with 125 engravings and 4 colored plates. Lea & Febiger, Philadelphia and New York, 1909. (Cloth, $3.25 net.)

Minor and Operative Surgery, Including Bandaging. By Henry R. Wharton, M. D., Professor of Clinical Surgery in the Woman's Medical College, Philadelphia. New (seventh) edition, enlarged and thoroughly revised. 12mo, 674 pages, with 555 illustrations. Lea & Febiger, Philadelphia and New York, 1909. (Cloth, $3.00, net.)

Progressive Medicine, Vol. III, September, 1909. A Quarterly Digest of Advances, Discoveries and Improvements in the Medical and Surgical Sciences. Edited by Hobart Amory Hare, M.D., Professor of Therapeutics and Materia Medica in the Jefferson Medical College of Philadelphia. Octavo, 336 pages, with 37 engravings. Lea & Febiger, Publishers, Philadelphia and New York. (Per annum, in four cloth-bound volumes, $9.00; in paper binding, $6.00, carriage paid to any address.)

A Textbook of Practical Therapeutics. By Hobart Amory Hare, M.D., Professor of Therapeutics in the Jefferson Medical College of Philadelphia. Thirteenth edition. Octavo, 951 pages, with 122 engravings, and 4 full-page colored plates. Lea & Febiger, Philadelphia and New York, 1909. (Cloth, $4.00; leather, $5.00, net; half morocco, $5.50, net.)

A Manual of Chemistry. A textbook specially adapted for students of medicine, pharmacy and dentistry. By W. Simon, Ph.D., M.D., Professor of Chemistry in the College of Physicians and Surgeons, Baltimore, and in the Baltimore College of Dental Surgery. Daniel Base, Ph.D., Professor of Chemistry in the Maryland College of Pharmacy. Ninth edition. Octavo, 716 pages, with 78 engravings and 9 colored plates, illustrating 64 of the most important chemical tests. Lea & Febiger, Philadelphia and New York, 1909. (Cloth, $3.00, net.)

The Principles of Bacteriology. A Practical Manual for Students and Physicians. By A. C. Abbott, M.D., Professor of Hygiene, University of Pennsylvania. Eighth edition. 12mo, 631 pages, with 100 illustrations, 26 in colors. Lea & Febiger, Philadelphia and New York, 1909. (Cloth, $2.75, net.)

Obstetrics. A Manual for Students and Practitioners. By David J. Evans, M.D., Lecturer on Obstetrics in McGill University, Montreal. Second edition. 12mo, 440 pages, with 169 illustrations. Lea & Febiger, Philadelphia and New York, 1909. (Cloth, $2.25, net.)

A Treatise on the Principles and Practice of Medicine. By Arthur R. Edwards, M.D., Professor of the Principles and Practice of Medicine and Clinical Medicine in the Northwestern University Medical School, Chicago. Second edition. Octavo, 1246 pages, with 100 engravings and 21 full-page plates in colors and monochrome. Lea & Febiger, Philadelphia and New York, 1909. (Cloth, $5.50, net; leather, $6.50, net.)

The Practical Medicine Series. Comprising ten volumes on the year's progress in medicine and surgery, under the general charge of Gustavus P. Head, M.D., Professor Laryngology and Rhinology at the Chicago Postgraduate Medical School. Vol. VI. General Medicine. Edited by Frank Billings and J. H. Salisbury, Chicago. Series, 1909. Chicago: The Year Book Publishers. (Price, $1.50.)

Physical Diagnosis. By Richard C. Cabot, Assistant Professor of Medicine in Harvard University. Fourth edition. 12mo, pp. 579. Illustrated. New York: William Wood & Co. (Cloth, $3.00.)

Parenthood and Race Culture. An Outline of Eugenics. By Calib Williams, Saleby, M.D., Ch.B., F.R.L., Edin., Fellow of the Obstetrical Society of Edinburgh, New York: Moffat, Yard & Co. (Cloth, $2.50.)

Medical Sociology. By James Peter Warbasse. 12mo, pp. 355. New York and London: D. Appleton & Co. (Cloth, $2.00.)

American Practice of Surgery. A complete System of the Science and Art of Surgery by representative surgeons of the United States and Canada. Edited by Joseph D. Bryant, M.D., and Albert H. Buck, M.D., New York. Complete in eight volumes. Vol. VI. Imperial octavo, pp. 916. Profusely illustrated. New York: William Wood & Company. 1909. (Price, cloth, $7.00.)

Practical Dietetics. By Gilman Thompson, Professor of Medicine in Cornell University Medical College. Fourth edition. Illustrated. Octavo, pp. 928. New York and London: D. Appleton & Co. (Cloth, $5.00.)

International Clinics. A Quarterly of Illustrated Clinical Lectures and especially prepared articles on Treatment, Medicine, Surgery, Neurology, Pediatrics, Obstetrics, Gynecology, Orthopedics, Pathology, Dermatology, Ophthalmology, Otology, Rhinology, Laryngology, Hygiene and other topics of interest to students and practitioners. Edited by W. T. Longcope, M.D., Volume III. Nineteenth series. Philadelphia and London: J. B. Lippincott Co. 1909. (Cloth, $2.00.)

Diagnostic Methods. Chemical, Bacteriological and Microscopical. By Ralph W. Webster, Assistant Professor of Pharmacological Therapeutics and Instructor in Medicine in Rush Medical College, University of Chicago. Illustrated. Octavo, pp. 641. Philadelphia: P. Blakiston's Son & Co. (Cloth, $6.00.)

Tuberculosis. Edited by Arnold C. Klebs. Illustrated. Octavo, pp. 939. New York and London: D. Appleton & Co. (Cloth, $6.00.)

The Principles of Hygiene. By D. H. Bergey, Assistant Professor of Bacteriology, University of Pennsylvania. Third edition. Illustrated. Octavo, pp. 555. Philadelphia and London: W. B. Saunders Co. (Price, $3.00.)

Medical Gynecology. By Samuel Wyllis Bandler, Fellow of the American Association of Obstetricians and Gynecologists, Adjunct Professor of Diseases of Women, N. Y. Post-Graduate Medical School and Hospital. Second edition. With original illustrations. Octavo, pp. 698. Philadelphia and London: W. B. Saunders Co. (Cloth, $5.00.)

Modern Materia Medica and Therapeutics. By A. A. Stevens, Professor of Therapeutics and Clinical Medicine in the Women's

Medical College of Pennsylvania. Fifth edition. Octavo, pp. 675. Philadelphia and London: W. B. Saunders Co. (Cloth, $3.50.)

A Textbook of Obstetrics. By Barton Cooke Hirst, Professor of Obstetrics in the University of Pennsylvania. Sixth edition. Illustrated. Octavo, pp. 992. Philadelphia and London: W. B. Saunders Co. (Cloth, $5.00.)

A Textbook of Surgical Diagnosis. For students and practitioners. By Edward Martin, M.D., Professor of Clinical Surgery, University of Pennsylvania, Philadelphia. Very handsome octavo of 764 pages, with 455 engravings, largely original, and 18 full-page plates. Lea & Febiger, Philadelphia and New York. (Cloth, $5.50, net.)

